# Latitudinal effects on phenology near the northern limit of figs in China

**DOI:** 10.1038/s41598-018-22548-7

**Published:** 2018-03-12

**Authors:** Huanhuan Chen, Yuan Zhang, Yanqiong Peng, Richard T. Corlett

**Affiliations:** 1 Center for Yunnan Plateau Biological Resources Protection and Utilization, College of Biological Resource and Food Engineering, Qujing Normal University, Qujing, Yunnan 655011 China; 2 Center for Integrative Conservation, Xishuangbanna Tropical Botanical Garden, Chinese Academy of Sciences, Menglun, Mengla, Yunnan 666303 China; 3 Yunnan Academy of Biodiversity, Southwest Forestry University, Kunming, 650224 China; 4 Key Laboratory of Tropical Forest Ecology, Xishuangbanna Tropical Botanical Garden, Chinese Academy of Sciences, Kunming, 650223 China

## Abstract

The interaction between pollinating wasps and figs is an obligate plant-insect mutualism, and the ca. 750 *Ficus* species are mainly tropical. Climatic constraints may limit species distributions through their phenology and this seems particularly likely for figs, where phenological mismatches can cause local extinction of the short-lived pollinators. We therefore compared the phenologies of *Ficus altissima*, *F. racemosa* and *F. semicordata* in tropical Xishuangbanna (21°55′N) and subtropical Liuku (25°50′N), SW China, to understand what factors limit fig distributions near their northern limits. All species produced synchronous crops of syconia in Xishuangbanna but production in Liuku was continuous, which may help maintain pollinator populations. However, in general, we found decreased fitness at the northern site: slower syconium development, so fewer crops each year; fewer seeds per syconium (two species); and fewer pollinators and more non-pollinators per syconium, so less pollen is dispersed. This is most easily explained by colder winters, although low humidities may also contribute, and suggests the northern limit is set by temperature constraints on reproductive phenology. If so, the warming predicted for future decades is expected to enhance the fitness of northern populations of figs and, in the longer term, allow them to shift their range limits northwards.

## Introduction

Climatic factors influence the phenology, physiology, distribution, and interactions of plant species, and climate change is altering these processes. Phenology may be the most sensitive of these to climate change^1^. Many studies in the northern temperate zone have shown that the onset of flowering has advanced and the growing season has shortened with warming^2,3^. Warming also increases the development rates of insects and causes shifts to higher altitudes and latitude^4^. Researchers are now investigating how climate change is causing, or not causing, species to change the timing of their cycles and their ability to survive in their present ranges and then using these data to predict their potential distribution areas in response to coming climate change^5–7^. This new perspective is forcing a rethinking of phenology’s place in ecological and evolutionary theory^8,9^.

Phenological shifts in mutualisms between flowering plants and insect pollinators caused by climate change can lead to mismatches between plant and pollinator populations that could potentially result in the extinction of both, with consequences for the plant-pollinator network^10,11^. Mismatches in pollination interactions have rarely been studied, however, and their demographic consequences are largely unknown^12^. Mutualism breakdown has the potential to expand and accelerate the impacts of global change on biodiversity and ecosystem functions^13^, and mutualist-mediated effects on species’ range limits have been proposed^14,15^. However, the mechanisms underlying the processes limiting species distributions are poorly understood. In some cases the limit may result from an inability to undergo full fruit ripening and/or flowering^16^.

The ca. 750 species of figs (*Ficus*, Moraceae) depend on short-lived (1–8 months, depending on temperature; 1–2 days as a winged adult), species-specific, wasp pollinators, which enter the closed, urn-shaped inflorescence (syconium). These wasps are, in turn, totally dependent on their host figs for reproduction, since their eggs can only develop in fig ovules. This mutualism is thus likely to be particularly vulnerable to phenological mismatches. In most species a suite of related non-pollinating fig wasps (NPFWs), usually detrimental to the fig’s reproductive success, also raise their offspring in the syconia^17,18^. Most non-pollinators are also apparently associated with a particular host species and thus also likely to be vulnerable to phenological mismatches. Most fig species are confined to the tropics, but some also extend into the subtropics, particularly in East Asia. The phenology of these species and their associated pollinating and non-pollinating fig wasps near their northern limits is therefore of particular interest.

Monoecious figs typically exhibit flowering synchrony at the individual level and asynchrony at the population level^19–21^, while dioecious figs, without the need to avoid inbreeding, often exhibit flowering asynchrony at the individual level^22^. The short-lived adult female pollinators depend on the availability of receptive syconia when they emerge, so the maintenance of the local pollinator population depends on a near continuous presence of receptive syconia, which permits a continuous cycling of pollinators between trees^23^. Although syconia can wait for a few weeks to be pollinated^24^, they are eventually aborted if no pollinators are available. Bronstein^23^ predicted that a median of 95 trees was required to produce an asynchronous sequence that would maintain local pollinator populations for 4 years in monoecious *Ficus natalensis*, under the assumption that adult female wasps can only survive for 1 day. Kameyama *et al*.^25^ suggested that the shorter intervals between flowering times in dioecious figs would allow smaller tree populations to maintain local pollinator populations.

China supports 125 fig species, of which 97 occur in the southwestern province of Yunnan^26^. The tropical prefecture of Xishuangbanna (22°00′N, 100°48′E) has the highest diversity in Yunnan, with 49 native species recorded from 19,700 km^2^. It is near the northern distribution limit for several fig species, so the fig/wasp mutualism is expected to be particularly vulnerable. Previous studies have found the abortion of figs due to the lack of pollinators and as well as prolonged receptive (B) phases waiting for pollinators^24^. Some pollinators arrive at host trees before their syconia are receptive, suggesting mismatches in phenology^27^. Yunnan is an excellent place to compare the maintenance of fig-fig wasp mutualisms across latitudes, as several species have a large latitudinal span, extending from the tropics north to the subtropical Nujiang (Salween River) region.

Reproductive phenology in figs has small seasonal fluctuations near the equator^23,28^. However, in the Xishuangbanna area, seasonal climate differences are significant, and figs generally produce more seeds and pollinators in the cool dry season and more non-pollinator fig wasps in the hot wet season^20^. The development period of figs is related to temperature^20^. The reproductive phenologies of figs and fig wasps are therefore affected significantly by climate. This study takes advantage of the fig diversity in Yunnan, and compares the phenology of both figs and their associated wasps in tropical Xishuangbanna and subtropical Liuku. We aimed to understand; (i) how figs and fig wasps respond to seasonal changes in climate along a latitudinal gradient; (ii) what factor(s) currently determine the northern limits of fig distributions in southwest China; and (iii) how climate change is likely to affect this mutualism.

## Results

### Weather in Xishuangbanna and Liuku

**Figure 1:**
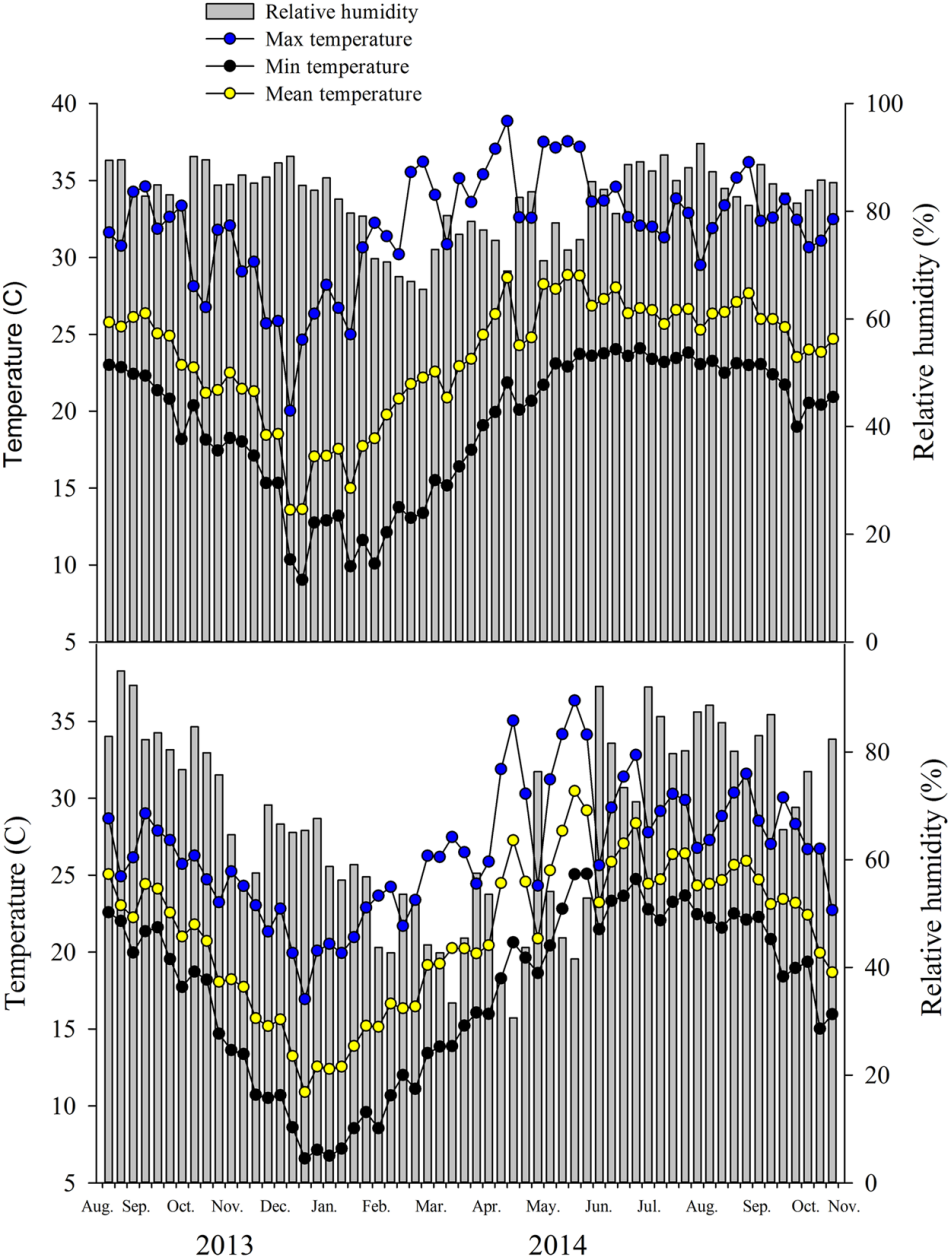
Maximum, mean, and minimum temperature and relative humidity measured in the canopies of fig trees in Xishuangbanna (top) and Liuku (bottom) during the study period.

Liuku was cooler and drier than Xishuangbanna during the study period, particularly in winter, with lower mean, minimum, and maximum temperatures, and lower relative humidity, particularly from January to June (Fig. 1). The lowest and highest temperatures recorded in Xishuangbanna in the study period were 6.4 °C and 40.5 °C and in Liuku were 4.6 °C and 37.5 °C. For relative humidities, the extremes were 65.5% and 92.6% in Xishuangbanna and 30.6% and 95.0% in Liuku.

#### Ficus altissima

*F. altissima* was briefly deciduous at both sites, but because flushing occurred immediately after leaf drop there were always some leaves on the trees (see Supplementary Fig. S1). The trees produced 2–3 flushes of new leaves each year, but small quantities of new leaves could also appear more or less continuously. Leaf initiation was not significantly correlated with either temperature or relative humidity in Xishuangbanna (Generalized Least Squares (GLS): temperature: *T* = 0.09, *P* = 0.93; relative humidity: *T* = 0.48, *P* = 0.63) or Liuku (temperature: *T* = 0.62, *P* = 0.95; relative humidity: *T* = −0.77, *P* = 0.44).

**Figure 2:**
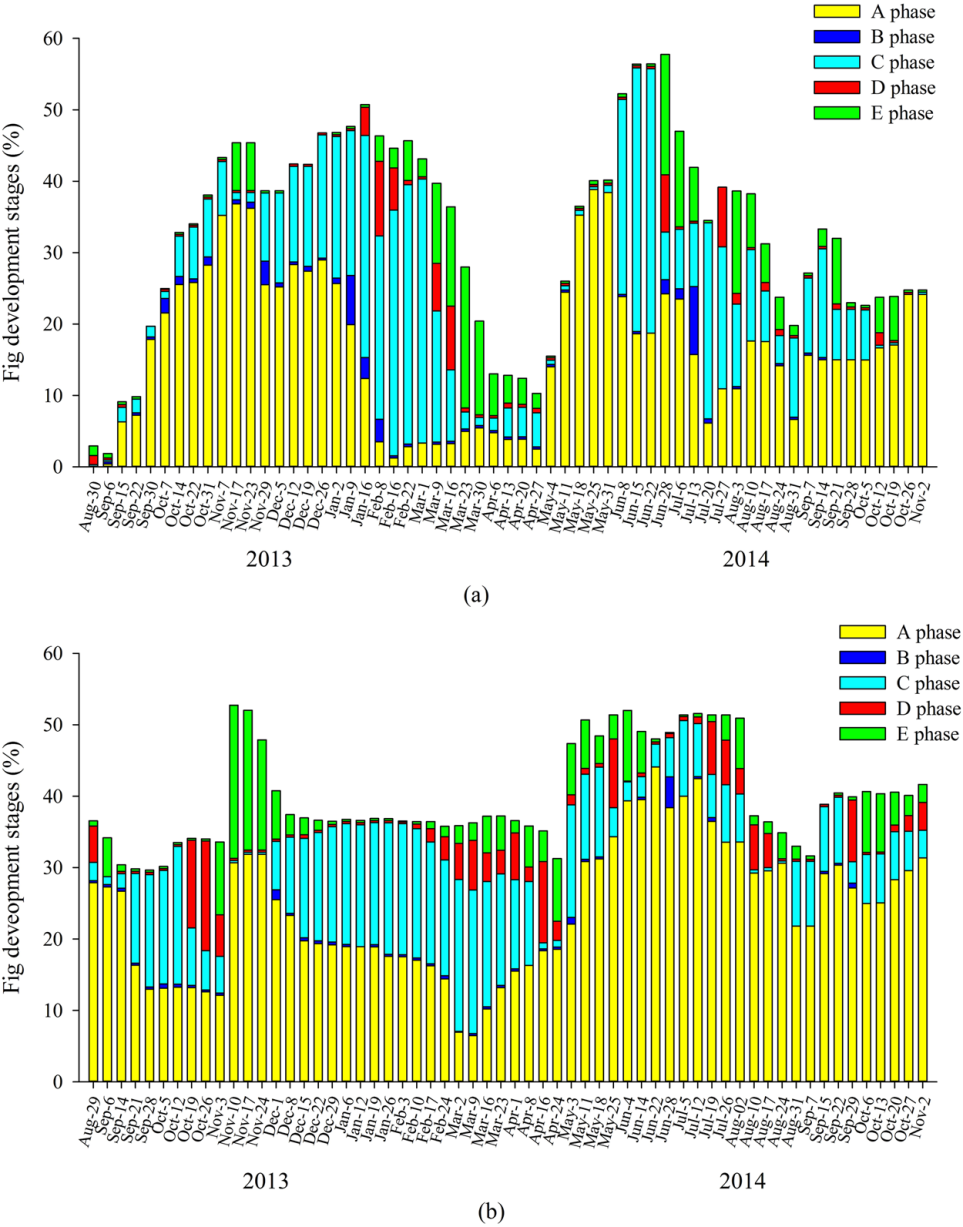
Annual reproductive phenology of *Ficus altissima* in (**a**) Xishuangbanna and (**b**) Liuku. A-E are successive stages in syconium development.

Syconia were present on *Ficus altissima* trees in Xishuangbanna all year around, with two distinct peaks, September-March and May-July (Fig. 2a). The mean development time of syconia in the summer wet season, when the mean temperature was 27.3 °C, was 62.6 days (range 50–85) days, while in winter, when the mean temperature was 19.2 °C, it was 150.7 days (95–176). Peak wasp emergence from D-phase syconia occurred February-March, from overwintering syconia, and in July, from syconia initiated in the wet season (Fig. 2a). There was some overlap between the crops of D phase and B phase syconia, particularly in winter, which would potentially allow adult fig wasps to find receptive syconia on the same tree (Supplementary Fig. S2a). At the population level, there were periods in September, November, and July when receptive syconia were not available on any of the sampled trees, but they were present on other trees in the surrounding area. Syconia initiation was significantly correlated with both temperature and relative humidity (temperature: *T* = 2.20, *P* = 0.03; relative humidity: *T* = 2.56, *P* = 0.01). Wasp emergence had no correlation with temperature (temperature: *T* = −1.08, *P* = 0.29).

Syconia were also present in Liuku at all times of the year, but initiation was continuous, with no well-defined crops, and development was asynchronous within trees (Fig. 2b). The development time in summer, when the mean temperature was 25.2 °C, was 107 days (78–141 days), while in winter, when the mean temperature was 18.9 °C, it was 255.5 days (252–259 days). There were more periods when emerging wasps would not find receptive syconia in the sampled population at Liuku, and fewer additional trees in the surrounding area, but this was potentially partly compensated by the asynchronous syconia production within trees and more overlap between phases (Supplementary Fig. S2b). Syconia initiation had no correlation with either temperature or relative humidity (GLS: temperature: *T* = 1.44, *P* = 0.16; relative humidity: *T* = 1.35, *P* = 0.18). Wasp emergence had no correlation with temperature (temperature: *T* < 0.01, *P* = 0.10).

In Xishuangbanna, the contents of the 183 syconia sampled from 9 crops were highly variable, with the mean number of seeds in different crops ranging from 15 to 300 and the mean number of female pollinators ranging from 13 to over 200 (Supplementary Table S1). Syconia containing more seeds also produced more pollinators (*T* = 15.08, *P* < 0.01). Cheaters (see Methods) were present in 7 crops in variable numbers. Overall, 11.5% of syconia contained only cheaters while 9.3% had both pollinators and cheaters. Cheaters both reduced the numbers of pollinator females within shared syconia (*T* = −6.07, *P* < 0.01) and had a significant negative impact on seed numbers (T = −28.14, *P* < 0.01).

In Liuku, the contents of the 134 syconia sampled from 7 crops were also highly variable, with the mean number of seeds in different crops ranging from 7 to 250 and the mean number of female pollinators ranging from zero to over 200 (Supplementary Table S1). Syconia had fewer female flowers in Liuku than in Xishuangbanna (*T* = 126.33, *P* < 0.01). Cheaters were present in all crops and in significantly higher numbers than in Xishuangbanna (*T* = 5.20, *P* < 0.01). Overall, 55.7% percent of syconia contained only cheaters and 17.1% had both cheaters and pollinators. The number of pollinators had a significant positive correlation with the number of seeds and a significant negative correlation with the number of cheaters (seeds: *T* = 110.61, *P* < 0.01; cheaters: *T* = −9.07, *P* < 0.01). Cheaters reduced the number of seeds within shared syconia (*T* = −8.90, *P* < 0.01). The numbers of seeds and pollinators were significantly lower in Liuku than in Xishuangbanna (seeds: *T* = 16.50, *P* < 0.01; pollinators: *T* = 8.36, *P* < 0.01).

In Xishuangbanna, in addition to the pollinator and cheater, there were 2–17 species in <9 genera of NPFW recorded from different crops (Supplementary Table S2). *Micranisa, Acophila, Philotrypesis, Sycophilomorpha* and *Sycoscapter* occurred in most of the crops, but in variable numbers. Genus *indet*, *Ormyrus*, *Sycobia* and *Sycophila* only occurred in some crops. In Liuku, there were 0–9 species (all also found in Xishuangbanna) in <6 genera. The diversity index of fig wasps in Xishuangbanna was 1.05 and evenness index 0.45 while in Liuku the diversity index was 0.76 and evenness index 0.32.

#### Ficus racemosa

*F. racemosa* was briefly deciduous at both sites, but there were always some leaves present because flushing occurred immediately after leaf drop (Supplementary Fig. S3). In Xishuangbanna, trees produced two major flushes of new leaves each year, from January to March and August to October, but small quantities of new leaves could also appear more or less continuously. At Liuku the trees produced a major flush of new leaves from February to March. New leaf initiation was correlated with both temperature and relative humidity in both Xishuangbanna (temperature: *T* = −3.61, *P* < 0.01; relative humidity: *T* = −3.50, *P* < 0.01) and Liuku (temperature: *T* = −4.28, *P* < 0.01; relative humidity: *T* = −4.48, *P* < 0.01).

**Figure 3:**
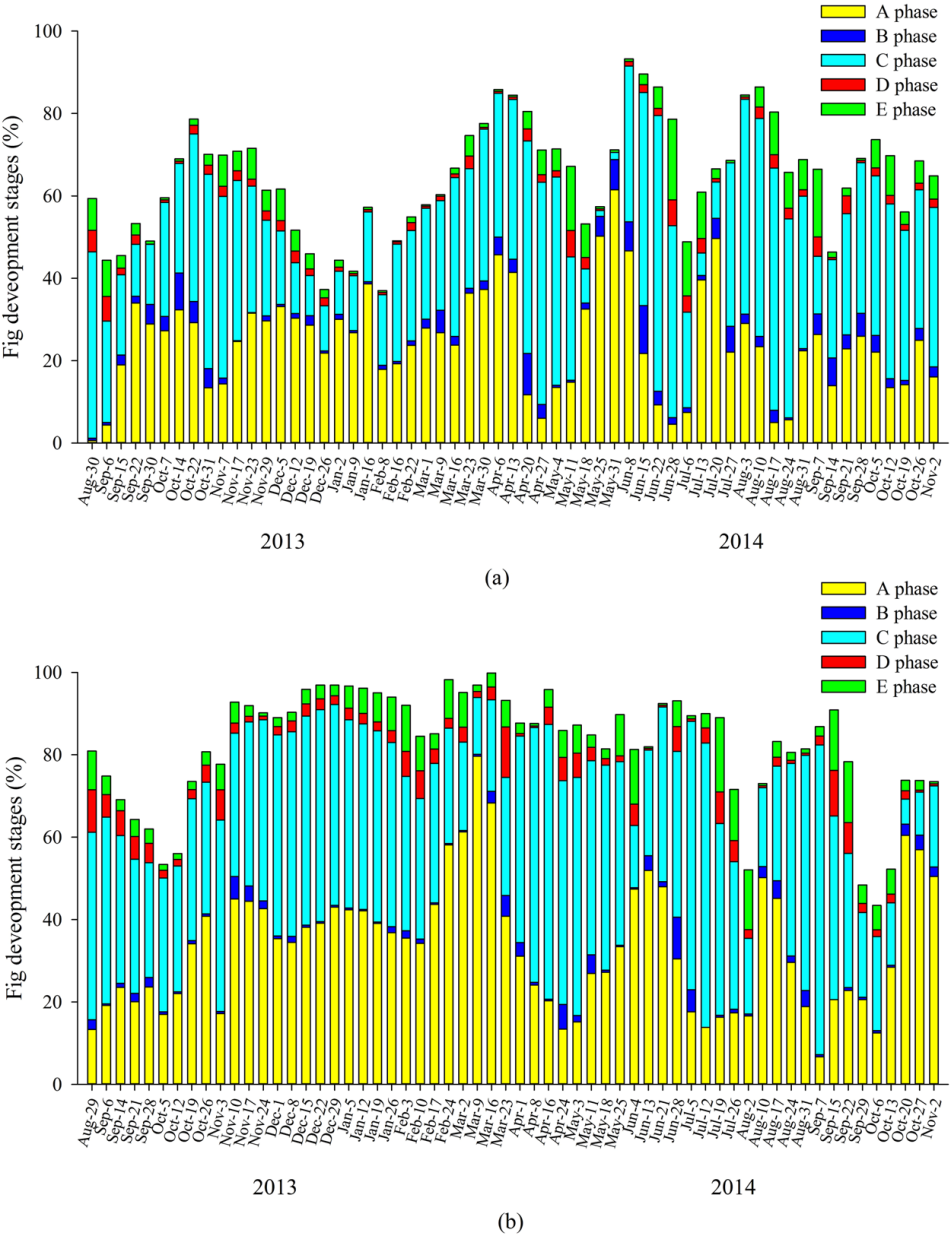
Annual reproductive phenology of *F. racemosa* in (**a**) Xishuangbanna and (**b**) Liuku. A-E are successive stages in syconium development.

Syconia were present on *Ficus racemosa* trees in Xishuangbanna at all times of the year, with 4–5 crops per year (Fig. 3a). Syconia development was synchronous within trees and asynchronous between trees. The mean development time in summer, when the mean temperature was 24.7 °C, was 49.7 days (range 34–80 days) and in winter, when the temperature was 17.9 °C, was 103.5 days (93–155). Peak wasp emergence from D-phase syconia occurred in February-March and July, but some wasps emerged throughout the year (Supplementary Fig. S4a). Syconia initiation had no correlation with either temperature or relative humidity (temperature: *T* = 1.37, *P* = 0.18; relative humidity: *T* = 1.21, *P* = 0.23),

Syconia were present year round in Liuku but development was asynchronous within trees (Fig. 3b). The mean development time in summer, when the mean temperature was 24.3 °C, was 58.7 days (36–82), and in winter, when the mean temperature was 16.1 °C, it was 129.5 days (128–134). Wasp emergence occurred year-round and pollination was also potentially facilitated by within-tree overlap between B and D phases, which was not recorded in Xishuangbanna (Supplementary Fig. S4b). Syconia initiation had no significant correlation with temperature or relative humidity (temperature: *T* = 0.03, *P* = 0.97; relative humidity: *T* = 0.22, *P* = 0.83).

In Xishuangbanna, the contents of the 174 syconia sampled from 11 crops were highly variable, with the mean number of seeds in different crops ranging from 156 to 4375, and mean number of female pollinators ranging from 8 to over 1855 (Supplementary Table S1). No crop was collected in January. The number of pollinators varied over the year. In Liuku, the contents of the 165 syconia sampled from 11 crops were also highly variable, with the mean number of seeds in different crops ranging from 0 to 1638, and the mean number of female pollinators from 1 to over 200. No crop was collected in February. The number of pollinators in Liuku was significantly lower than in Xishuangbanna (*T* = 25.27, *P* < 0.01) and none were collected from some crops. The numbers of seeds and female flowers were also significantly lower than in Xishuangbanna (seeds: *T* = 25.00, *P* < 0.01; female flowers: *T* = 114.81, *P* < 0.01).

In addition to pollinators, 5 species of NPFWs were recorded at both sites, *Platyneura testacea*, *P. mayri*, *P. agraensis*, *Apocrypta westwoodi*, and *A*. sp. 2., with large variation in the numbers of individuals (Supplementary Table S3). The proportion of NPFWs in Liuku was high, especially in winter, when NPFWs were sometimes trapped in syconia because no male pollinators were available to excavate a passage for the wasps to emerge. This was seen more rarely in Xishuangbanna, where pollinators were usually dominant. As a result, the diversity index of fig wasps in Xishuangbanna was 0.91 and evenness index 0.51, while the diversity index in Liuku was 1.33 and the evenness index 0.74.

#### Ficus semicordata

The dioecious *F. semicordata* in Xishuangbanna is evergreen, but leaf replacement was discontinuous and showed no relationship with syconia production (Supplementary Fig. S5). Male and female trees produced one major flush of new leaves in March each year, but small quantities of new leaves also appeared more or less continuously. In both Xishuangbanna and Liuku, leaf initiation had no correlation with temperature in either male or female trees (Xishuangbanna male trees: *T* = −0.52, *P* = 0.60; female trees: *T* = −0.42, *P* = 10.71; Liuku male trees: *T* = 0.64, *P* = 0.53, female trees: *T* = 0.55, *P* = 0.61), and also none relative humidity (Xishuangbanna male trees: *T* = −0.77, *P* = 0.45; female trees: *T* = −0.51, *P* = 0.37; Liuku male trees: *T* = −0.38, *P* = 0.70; female trees: *T* = −0.42, *P* = 0.60).

**Figure 4:**
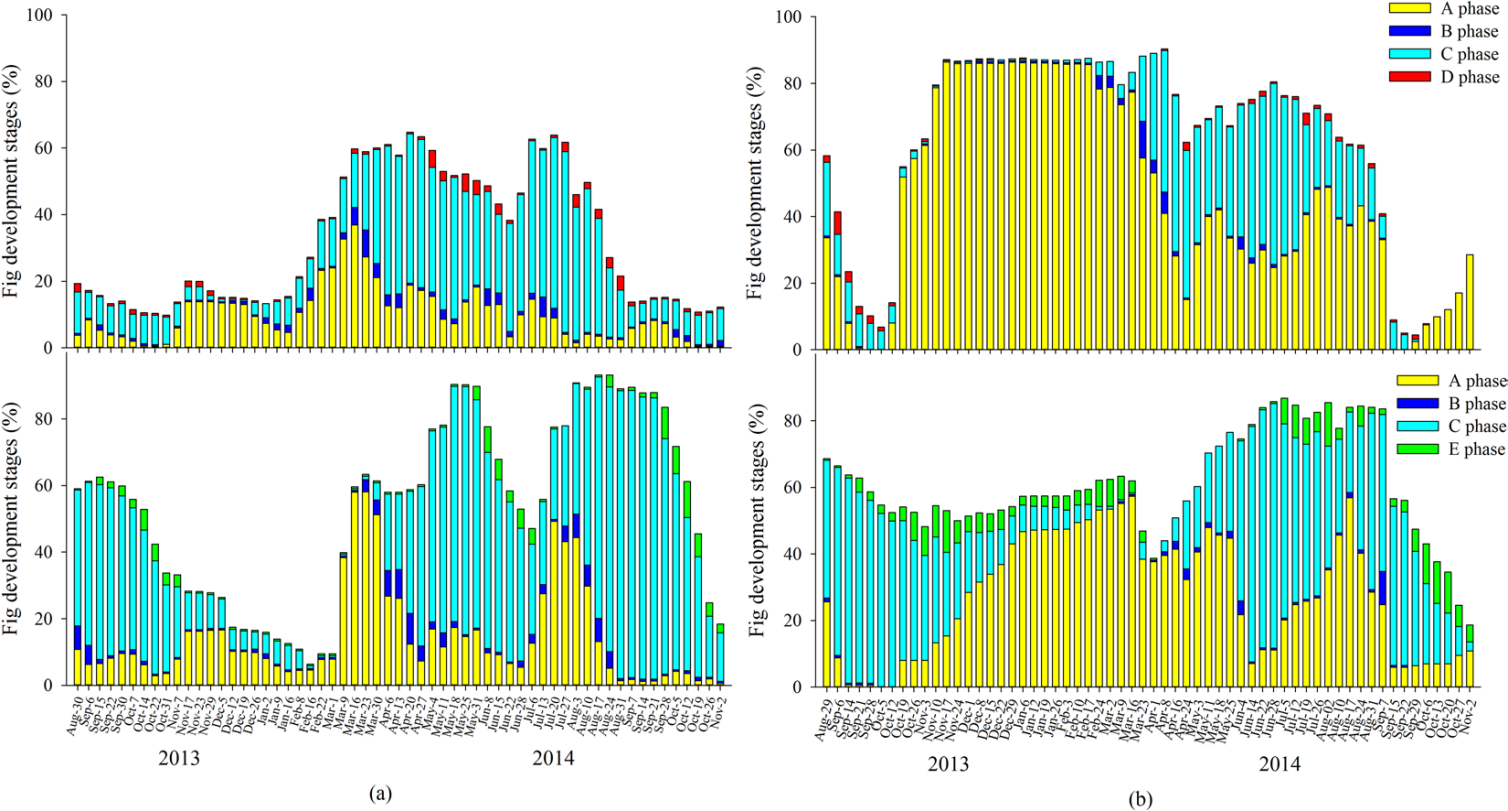
Annual reproductive phenology of *F. semicordata* in (**a**) Xishuangbanna and (**b**) Liuku. A-E are successive stages in syconium development.

Both male and female trees of *Ficus semicordata* in Xishuangbanna produced 2–3 crops in the warmer season (Fig. 4a). Wasp production by D-phase syconia generally matched well with the presence of receptive B-phase syconia (Supplementary Fig. S6a). Male trees also initiated a few syconia in winter. The mean development time of male syconia in summer, when the mean temperature (during the period when the male trees bore crops) was 24.0 °C, was 79.5 days (50–105), while in winter, when the mean temperature was 18.4 °C, it was 145.5 days (144–147 days). The mean development time of female syconia in summer, when the mean temperature (while the female trees bore crops) was 23.9 °C, was 104.2 days (55–154). Syconia initiation had no significant correlation with either temperature or relative humidity in male trees (temperature: *T* = 0.46, *P* = 0.64; relative humidity: *T* = −0.72, *P* = 0.47) or female trees (temperature: *T* = 0.31, *P* = 0.55; relative humidity: *T* = −0.42, *P* = 0.51).

Syconia of *Ficus semicordata* in Liuku were produced continuously with no well-defined crops (Fig. 4b). A-phase syconia produced in winter by both sexes aborted due to lack of pollinators, with new small syconia initiated soon after, while those initiated after March in the male trees developed to maturity (Supplementary Fig. S6b). The mean development time of male syconia in summer, when the mean temperature in male trees was 22.9 °C, was 96.7 days (177–113), while the mean development time of male syconia in winter when the mean temperature was 17.1 °C, was 196.5 days (182–213). The mean development time of female syconia in summer, when the mean temperature was 20.9 °C, was 127 days (102–156). Syconia initiation had a significant positive correlation with both temperature and relative humidity in both male trees (temperature: *T* = 4.32, *P* < 0.01; relative humidity: *T* = 2.74, *P* < 0.01) and in female trees (temperature: *T* = 3.56, *P* < 0.01; relative humidity: *T* = 2.93, *P* < 0.01). Wasp emergence was also positively correlated with temperature (*T* = 3.06, *P* < 0.01).

In Xishuangbanna, the contents of the 136 syconia sampled from 9 male crops were highly variable, with the mean number of female pollinators ranging from 16 to over 621 (Supplementary Table S1). In Liuku, the 104 syconia sampled from 7 male crops were also highly variable, with mean number of female pollinators ranging from 17 to over 448. The mean numbers of pollinators and seeds were significantly higher in Xishuangbanna than in Liuku (pollinator: *T* = 31.32, *P* < 0.01; seeds: *T* = 49.94, *P* < 0.01). In addition to the pollinator, there were four species of NPFWs recorded from different crops at both sites, with large variation in the numbers of individual species (Supplementary Table S4). The diversity index of fig wasps in Xishuangbanna was 0.85 and the evenness index 0.53, while in Liuku the diversity index was 1.00 and the evenness 0.62.

## Discussion

Xishuangbanna (21°55′N) is near the northern margin of tropical SE Asia, but has a very diverse local fig flora. Despite its northerly location and strongly seasonal climate, the reproductive phenologies of *F. altissima* and *F. racemosa* in Xishuangbanna are similar to those described for most other monoecious *Ficus* species, with flowering synchrony at the individual level and synchrony at the population level^23,29,30^. Three monoecious fig species near the poleward margins of their ranges in southern Brazil, in similar climates to Xishuangbanna, also had aseasonal or weakly seasonal phenologies^31,32^. Dioecious figs have more varied phenologies, but that of *F. semicordata* in Xishuangbanna is fairly typical.

Subtropical Liuku (25°50′N), in contrast, although only a few degrees cooler, is near or beyond the northern limits of distribution for many fig species. Syconia development is strikingly longer than in Xishuangbanna in the two monoecious species studied and syconia production was no longer synchronized at the tree level. This loss of synchrony is probably adaptive, since it could permit within-tree cycling of pollinators and potentially the production of selfed seeds, if there are no other receptive trees in the vicinity, while also increasing the likelihood that the D and B phases will overlap with the appropriate phases of other trees^33^. We did not test if loss of synchrony permitted seed production by selfing in our species, but even if selfing does not occur, loss of synchrony may help in the maintenance of local pollinator populations in both monoecious and dioecious figs^34^.

Liuku also has a much lower humidity in winter and spring than Xishuangbanna, and this may reduce the survival time of flying female pollinators^35^. However, summer humidities are similar at the two sites, while differences in phenology are evident year-round, suggesting that temperature rather than humidity is the most important factor. Moreover, wasp larvae inside syconia are shielded from low humidities but not temperatures. A key role for winter temperatures is also consistent with other observations of fig phenologies outside the tropics.

Both monoecious species produced fewer seeds and pollinators per fig in Liuku, and cheaters were much more abundant in *F. altissima* than in Xishuangbanna (Supplementary Table S1). *F. semicordata* also produced fewer pollinators and the receptive syconia produced in winter were often aborted because they were not pollinated. Latitudinal differences in non-pollinating fig wasps, in contrast, had no consistent patterns. There were more species in Xishuangbanna than Liuku in *F. altissima*, perhaps reflecting the rarity of this fig species at the northern site, but the numbers of species were the same at both sites for the other two fig species. In *F. racemosa* NPFWs were particularly abundant in winter, when they were sometimes trapped inside the syconia by the absence of the male pollinators needed to excavate a passage to the outside.

One monoecious fig species, *F. virens*, extends north of Yunnan to at least 30°29′N in Sichuan Province^36^. Even at this latitude, crops were synchronized within trees and asynchronous within the population, but very few trees retained syconia in winter, when nighttime temperatures can fall below freezing, and syconia development was very slow. Despite this, most spring syconia were pollinated, suggesting that pollinators may be dispersing from further south. The population also supported at least 10 genera of NPFWs. At a similar latitude (31°45′N) in Sichuan, the phenology of dioecious *F. tikoua* showed a strong convergence with that of other, unrelated, temperate dioecious figs (*F. pumila* and *F. erecta* in eastern China, and *F. carica* in Europe), with relatively synchronized crops and only male plants bearing syconia over winter^37^.

Even in the best-adapted fig species, however, slowing development times for syconia in colder weather must eventually reduce fitness. A simulation study by Kameyama *et al*.^25^ showed that short intervals between crops mean that fewer fig plants are required to maintain a viable wasp population, by reducing the chance of a gap in the population-level availability of receptive syconia. The longest development times recorded in this study were for *F. altissima*, where crops initiated in August in Liuku took more than 8 months to mature. Of the three species studied, this species is nearest its northern limits in Liuku and it was also the only one to show a decline in the number of NPFWs recorded.

The different sizes, life spans, and mobilities of the partners, coupled with interannual variation in climate, would be expected to produce a ragged edge at the poleward limits of the range, where fig plants survive occasional or annual winters too cold for the maintenance of a local pollinator population, and the wasps recolonize from nearer the tropics—or perhaps from local refuges with a favorable microclimate—in spring. The pollinators of monoecious figs have been shown to disperse as much as 160 km^38^ and genetic evidence suggests that similar dispersal abilities occur in the pollinators of at least some dioecious figs^39,40^. Unusual cold extremes often kill the leaves or all the above-ground parts of fig plants near their climatic limits, but in China the fig species that occur in the subtropics show a striking ability to resprout from surviving woody parts, so the set-back is only temporary (personal observation in Kunming by HC and >500 m a.s.l. in Hong Kong by RTC). In *Ficus hirta*, genetic evidence from plant and pollinator populations suggests that the fig plants may survive climatic extremes that their pollinators do not, with the latter recolonizing from refuges further south^39^.

If low temperatures are the major limiting factor near the poleward limits of figs, then anthropogenic climate change would be expected to extend these limits. Indeed, most of Yunnan has already warmed considerably in the last 50 years, particularly in winter and spring, and particularly since the 1980s^41^. This may already have improved the fitness of northern fig populations. A further 1.6 to 4.8 °C warming is expected by 2050, depending on the assumptions made, but trends in rainfall are unclear^42^. This projected warming is expected to further enhance the fitness of currently marginal populations and permit their expansion to higher latitudes and altitudes. Latitudinal expansion is likely to be limited by the dispersal of seeds rather than pollinators. *F. altissima* is bird-dispersed and fruiting trees appear to be highly attractive to frugivorous species, but the dispersal agents for the large figs of *F. racemosa* and the smaller, partly buried, figs of *F. semicordata* in Yunnan are unknown. Elsewhere, *F. racemosa* is dispersed by large fruit bats which are absent from Yunnan^40^.

The fig/fig-wasp mutualism is the center of a diverse web of interacting species, including NPFWs, other invertebrate inhabitants of developing syconia, such as nematodes, and vertebrate frugivores that consume the ripe figs. This study and the others mentioned above show that most monoecious fig species continue to support a diverse fauna of NPFWs near the poleward limits of their range, and a study on the island of Yakushima, Japan, found that the monoecious *Ficus superba* still attracted a diverse frugivore assemblage year-round at 30°N^43^. Dioecious figs support fewer NPFWs, are mostly smaller plants with smaller crops, and do not supply a year-round source of ripe figs in seasonal climates, but their densities in some areas can be high, so they may be a seasonally significant resource for frugivores^7^.

## Conclusions

The results of this study are consistent with the idea that phenology can play an important role in determining the range limits of plant species, and suggest that changes in phenology, as a result of plasticity or genetic variation, have allowed some tropical fig species to extend their ranges polewards. Phenology is strongly constrained in monoecious figs, because of the need to maintain local pollinator populations, but dioecious figs have greater flexibility and a few species extend into warm temperate climates. Conversely, an inability to change phenology appears to limit the ranges of many fig species, monoecious and dioecious, to the tropics.

## Methods

### Study sites

**Figure 5:**
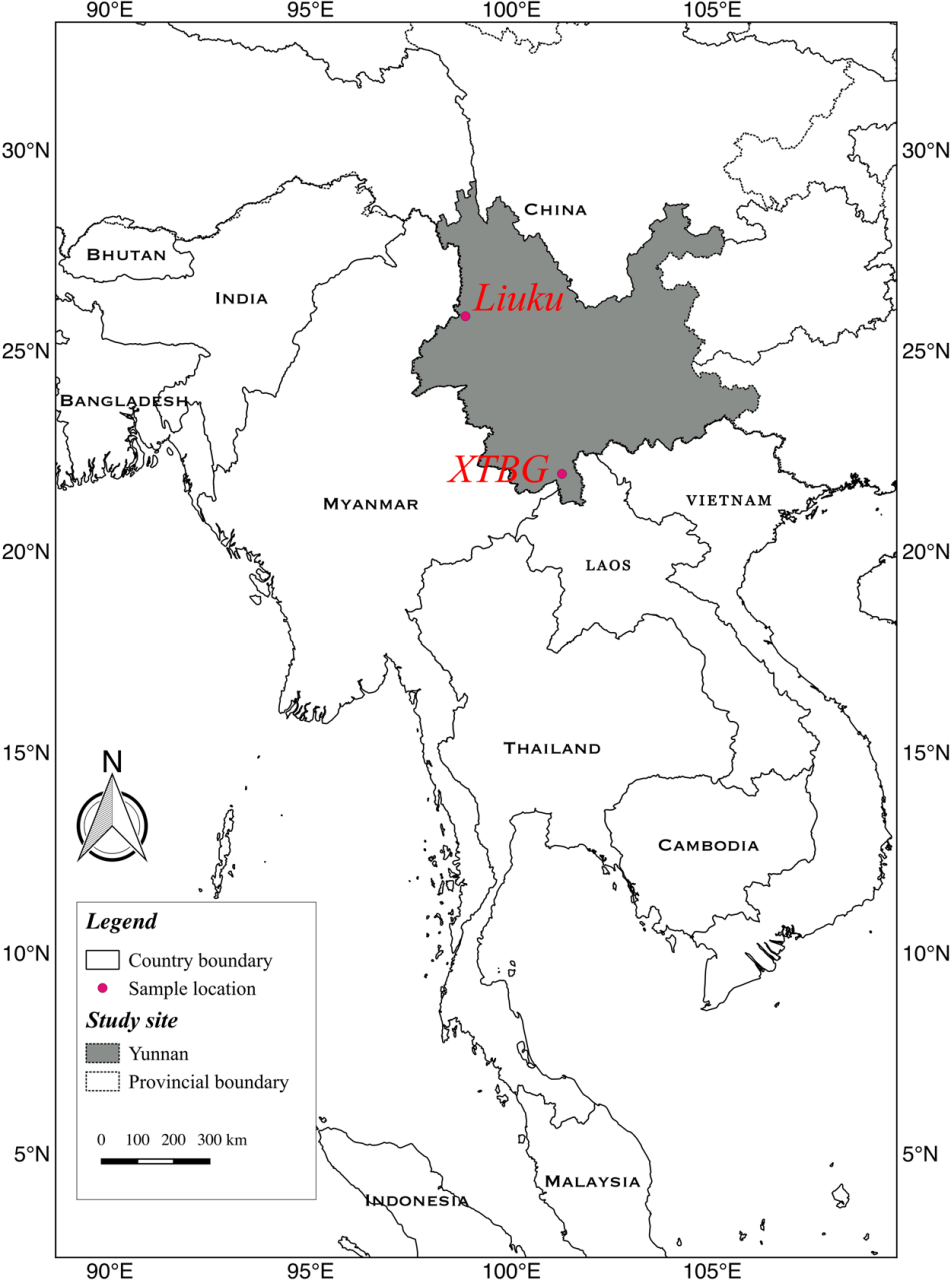
Locations of the study sites, Xishuangbanna and Liuku, in northern tropcial and subtropical East Asia. The map was generated from vector data in the public domain provided by Natural Earth (http://www.naturalearthdata.com/about/terms-of-use/) and was completed with the open-source software QGIS 2.14 (http://www.qgis.org/en/site/getinvolved/governance/trademark).

This study was carried out in and around the Xishuangbanna Tropical Botanical Garden (XTBG; 101^◦^15′E, 21^◦^55′N) in SW Yunnan, China, near the northern margin of tropical SE Asia, and at Liuku (98^◦^51′E, 25^◦^50′N), in subtropical NW Yunnan (Fig. 5). Xishuangbanna has a tropical monsoon climate, with a mean annual rainfall of 1500 mm, mean annual temperature of 23.2 °C, and means of 30.6 °C in the hottest month (June) and 16.0 °C in the coldest month (January). It experiences three main seasons: a foggy cool season (November to February), a dry hot season (March to April) and a rainy season (May to October). The Xishuangbanna study area is 554 m above sea-level. Liuku is drier and cooler than Xishuangbanna, with a mean annual rainfall of 1115 mm, mean annual temperature of 21.1 °C, and means of 26.8 °C in the hottest month (June) and 13.1 °C in the coldest month (December). The Liuku study area is 828 m above sea-level, which accounts for part of the difference in temperature from Xishuangbanna. XTBG has reliable long-term weather records that show a significant warming trend of 0.13 °C per decade and an insignificant trend of increasing rainfall of 46 mm per decade for the last 40 years^44^. Long-term records from single rural weather stations can be less reliable, but quality controlled data for the region around Liuku show the same warming trend as at XTBG^45^ and, in contrast to XTBG, a significant trend of decreasing rainfall of 27 mm per decade^46^.

### Species biology

*Ficus altissima* is a large hemiepiphytic monoecious fig species belonging to subgenus *Urostigma* section *Conosycea*. Its native range covers tropical and subtropical areas of much of Asia^47^. It is pollinated by *Eupristina altissima*, but also supports an undescribed congener (‘cheater’ *Eupristina* sp.) which has reduced pollen pockets and fails to pollinate, but enters figs at the same developmental stage and has similar biology^48^ *F. altissima* also supports at least 25 species of non-pollinating fig wasps (NPFW) locally, including ovule gallers and parasitoids^20,49^, but their detailed biology is largely unknown.

*Ficus racemosa* is a monoecious large tree, belonging to primarily dioecious subgenus *Sycomorus* section *Sycomorus*, and has a native distribution covering much of tropical Asia through to Australia^47^. It bears cauliflorous syconia. The pollinating wasps are *Ceratosolen fusciceps*, while NPFWs recorded from the syconia include *Sycophaga testacea*, *Sycophaga mayri*, *Sycophaga agraensis*, *Apocrypta westwoodi* and *Apocrypta* sp.^50^.

*Ficus semicordata* is a dioecious small to medium tree, belonging to subgenus *Sycomorus* section *Hemicardia*, and is widely distributed in the northern tropics and subtropics of Asia^47^. Fig-bearing branches are near to roots or go into the ground. The pollinating wasps are *Ceratosolen gravelyi*, while NPFWs recorded from the syconia include *Sycophaga cunia*, *Sycoscapter trifemmensis*, *Philotrypesis dunia*, and *Apocrypta* sp.^51,52^.

### Phenological censuses

We made phenological observations of the same three fig species at each study site at weekly intervals from August 2013 to November 2014. In Xishuangbanna, the individual trees observed were only a small part of larger populations within a few kilometers of the study area. This was also true for *Ficus semicordata* in Liuku, but the other two species, *F. altissima* and *F. racemosa*, were relatively rare at this site and the individual trees observed were all or almost all of the mature trees we could find in the area. We observed 12 *F. altissima* in Xishuangbanna and 11 in Liuku, 23 *F. racemosa* in Xishuangbanna and 10 in Liuku, and 14 male and 14 female trees of *F. semicordata* in Xishuangbanna and 12 male and 10 female trees in Liuku. Individual trees were tens of meters to several kilometers apart. The presence or absence of young (newly expanding out of buds), growing, mature, and senescing leaves, and the numbers and developmental phases of syconia (figs), were recorded at each visit. Five fig developmental phases were distinguished, modified from Galil and Eisikowich^53^: pre-female phase (A), female phase (B), interfloral phase (C), male phase (D, on male plants only in *F. semicordata*) and post-floral phase (E, on female plants only in *F. semicordata*). We measured the mean development time of crops from fist appearance of the the syconia (the start of phase A) to ripeness (stage E or, for male crops, the end of stage D) for crops developed in the wet summer season, from May to October, and in the cool winter season from November to April. Thirty D-phase syconia per tree were collected. Each syconium was placed individually in a fine-mesh bag (20 × 20 cm) and the fig wasps allowed to emerge. All wasps, including those remaining inside the syconia, were collected and preserved in 75% ethanol. For 15 syconia of *F. racemosa*, 15 male and 15 female syconia of *F. semicordata*, and 30 of the smaller syconia of *F. altissima*, all male flowers, seeds, bladders, unemerged wasps, and other female flower fates were counted.

Temperature and relative humidity were recorded by Onset HOBO data loggers (U23–001) in three individual trees of each species at each site at half an hour intervals throughout the study period. The temperature and humidity records from the HOBOs during study period were very highly correlated with those from the nearest weather stations (Xishuangbanna, temperature: R = 0.99, humidity: R = 0.95; Liuku, temperature: R = 1.0, humidity unavailable). Rainfall and temperature seasonality at both weather stations were also very similar during the study period (2013–2014) to the means for the last 10 years (Supplementary Fig. S7).

### Data analysis

The proportions of trees with new (expanding) leaves and new syconia (newly visible A phase syconia) were calculated after every census, and related to mean temperature and relative humidity during the preceding week using Generalized Least Squares (GLS). Temporal autocorrelation is likely for leaf initiation, syconia initiation, and wasp emergence, so we estimated this with the autocorrelation function (ACF) and included all lagged periods with significant influence in the final model. Correlation coefficients between pollinators and seeds, between cheaters and pollinators, and between cheaters and seeds and fig contents at the two sites were compared with GLM. All analyses were conducted in R 3.1.2 Professional Version. Fig wasp community diversity for the three *Ficus* species at each site was calculated using Shannon-Wiener’s diversity index (${\rm{H}}^{\prime} =-{\sum }_{{\rm{i}}}^{{\rm{S}}}{\rm{Piln}}({\rm{pi}}),$ where P_i_ is the proportion of individuals belonging to the *i*th species, S is the number of fig wasp species) and Pielou’s evenness (E = H′/LnS, where S is the number of fig wasp species).

## Electronic supplementary material

Supplementary Figures and Tables
