# Supplementary material for: Latitudinal effects on phenology near the northern limit of figs in China
Source: Sci Rep. 2018 Mar 12;8:4320. doi: 10.1038/s41598-018-22548-7 (PMC5847597; doi:10.1038/s41598-018-22548-7)
Supplement: Supplementary file 1 — Supplementary Figures and Tables [file 41598_2018_22548_MOESM1_ESM.pdf]

Supplementary Information for 'Latitudinal effects on phenology near the northern limit of figs in China.'

Huanhuan Chen[1, 2], Yuan Zhang[3], Yanqiong Peng[4*] & Richard T. Corlett[2*]

[1]Center for Yunnan Plateau Biological Resources Protection and Utilization, College of Biological Resource and Food Engineering, Qujing Normal University, Qujing, Yunnan 655011, China

[2]Center for Integrative Conservation, Xishuangbanna Tropical Botanical Garden, Chinese Academy of Sciences, Menglun, Mengla, Yunnan 666303, China.

[3]Yunnan Academy of Biodiversity, Southwest Forestry University, Kunming 650224, China.

[4]Key Laboratory of Tropical Forest Ecology, Xishuangbanna Tropical Botanical Garden, Chinese Academy of Sciences, Kunming 650223, China.

Correspondence and requests for materials should be addressed to R.T.C. (email: corlett@xtbg.org.cn) and P. Y. Q. (email: pengyq@xtbg.ac.cn)

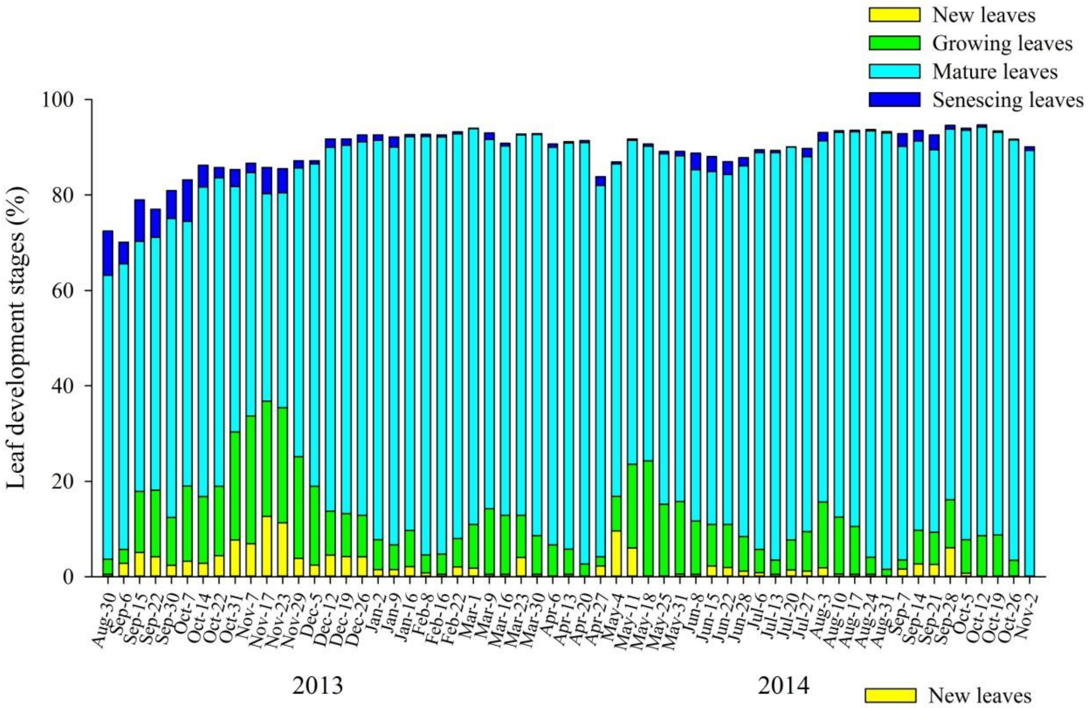

(a)

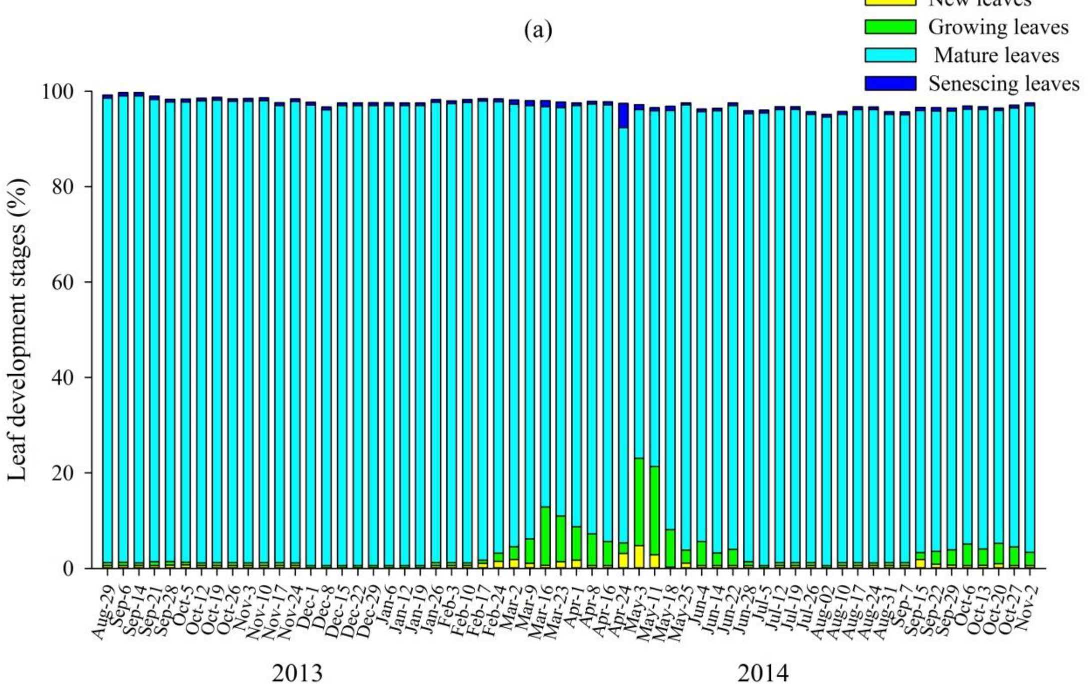

(b)

Supplementary Figure S1. Annual leaf phenology of *F. altissima* in (a) Xishuangbanna and (b) Liuku.

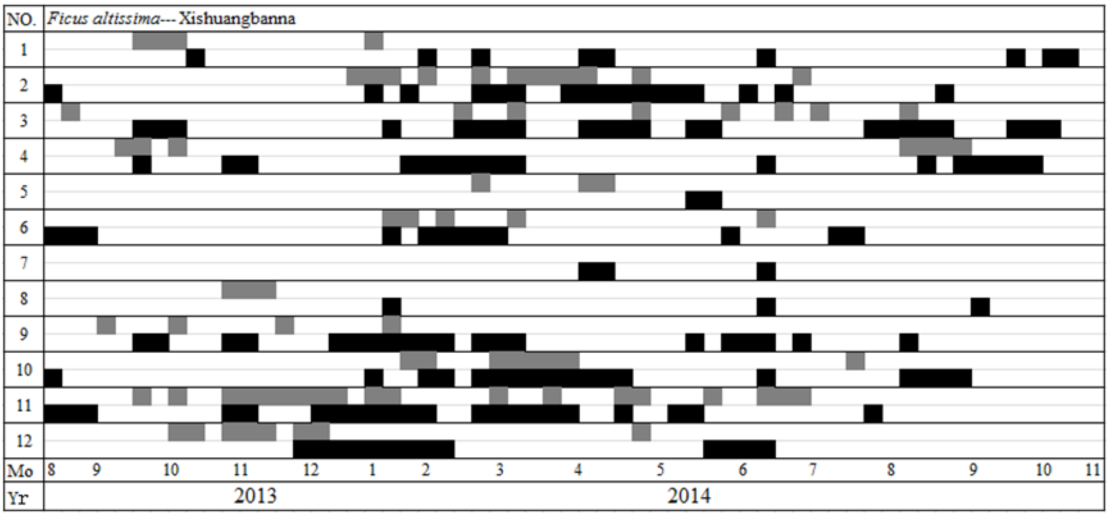

(a)

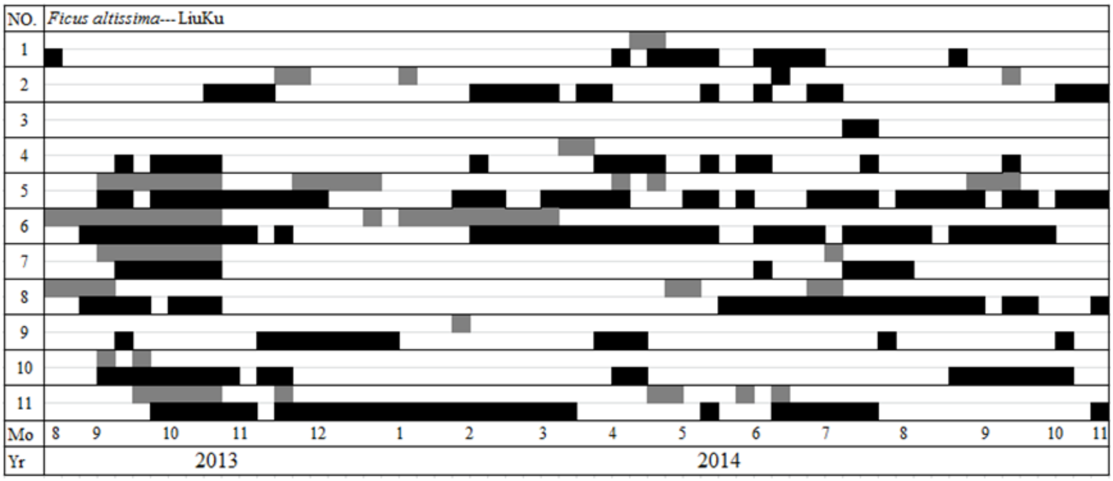

(b)

Supplementary Figure S2. Production of B phase (grey) and D phase (black) syconia in *F. altissima* in (a) Xishuangbanna and (b) in Liuku.

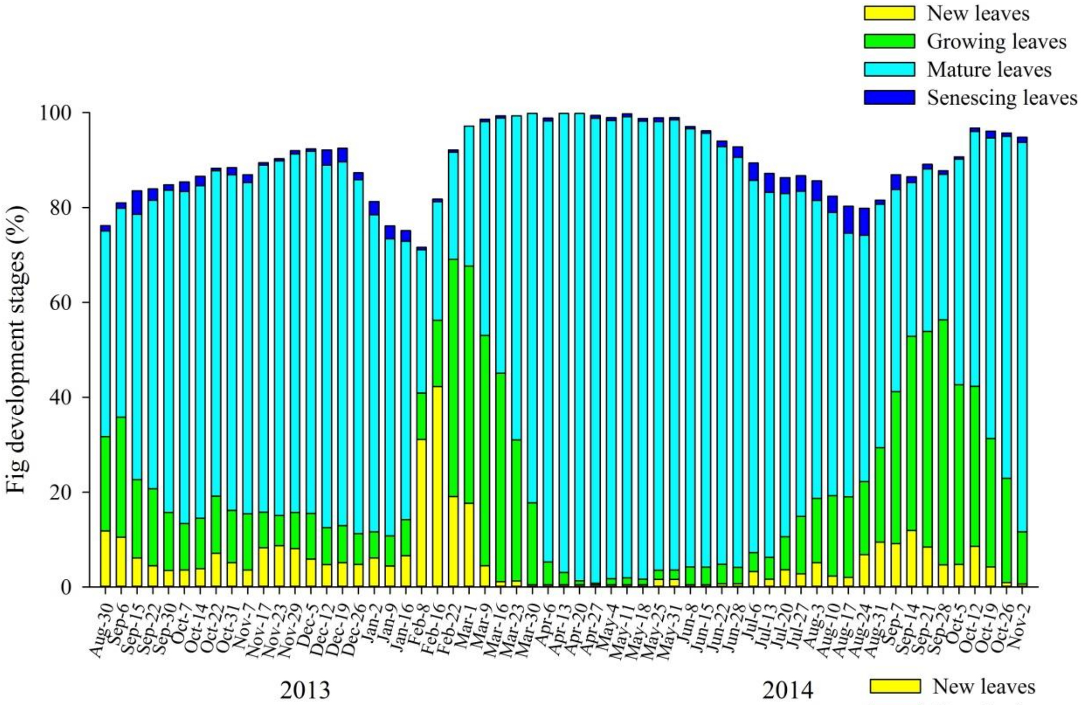

(a)

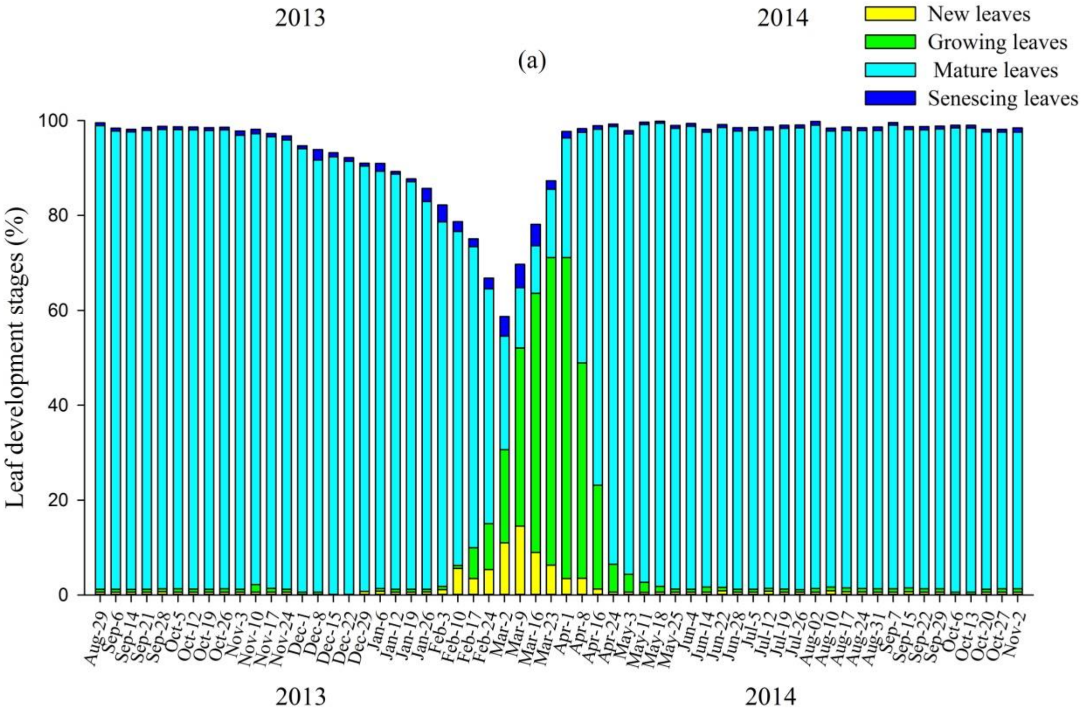

(b)

Supplementary Figure S3. Annual leaf phenology of *F. racemosa* in (a) Xishuangbanna and (b) Liuku.

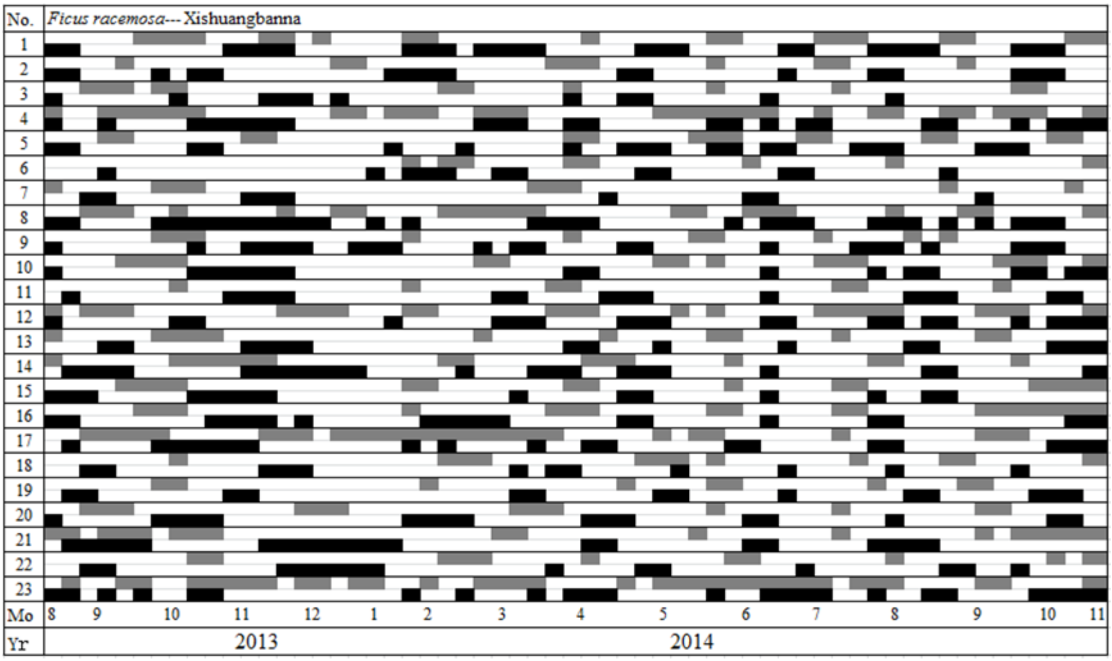

(a)

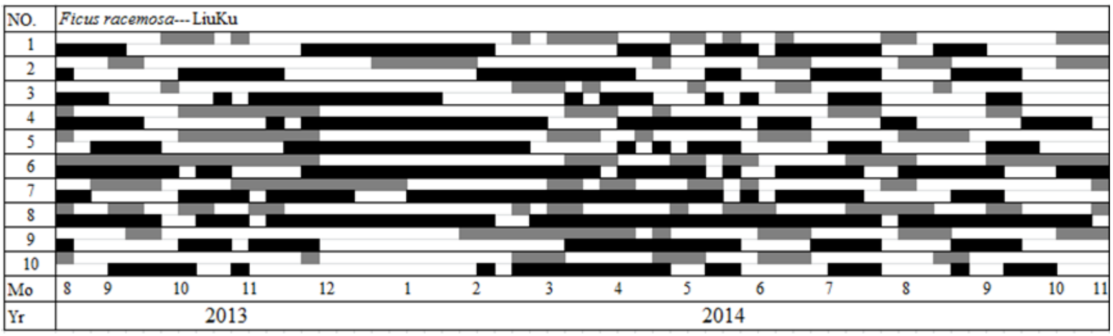

(b)

Supplementary Figure S4. Production of B phase (grey) and D phase (black) syconia in *F. racemosa* in (a) Xishuangbanna and (b) in Liuku.

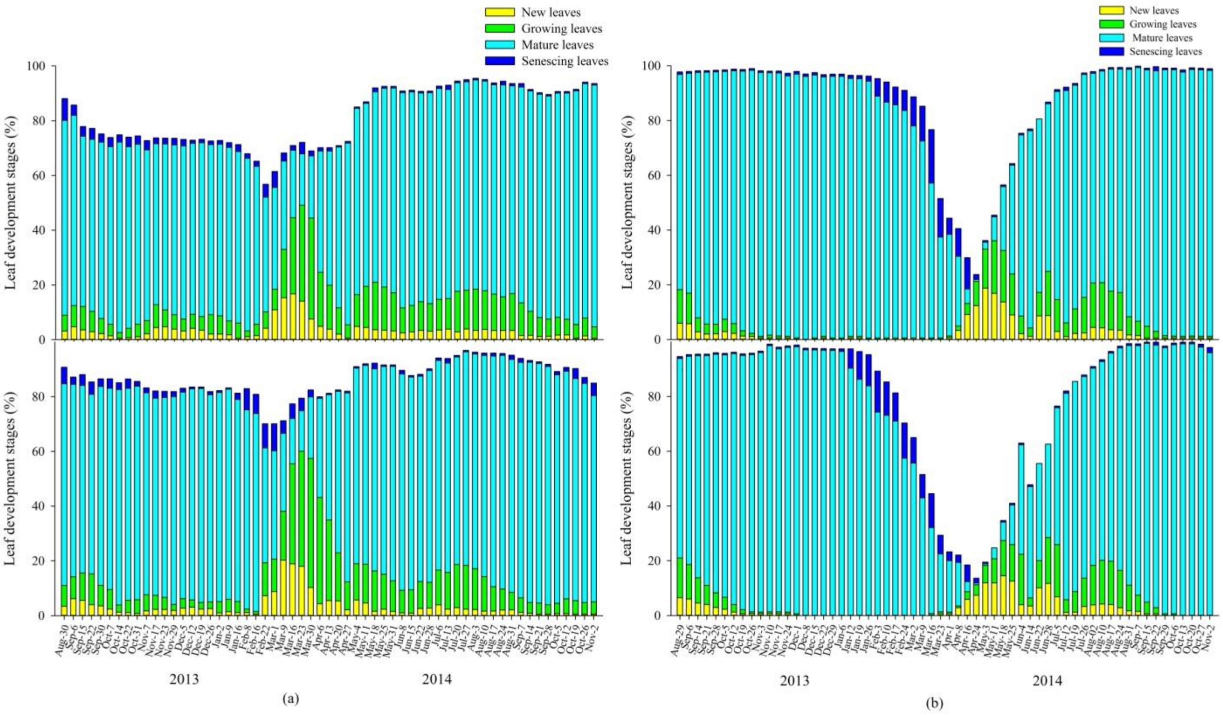

Supplementary Figure S5. Annual leaf phenology of *F. semicordata* in (a) Xishuangbanna and (b) Liuku. Top row are male plants, bottom row are female plants.

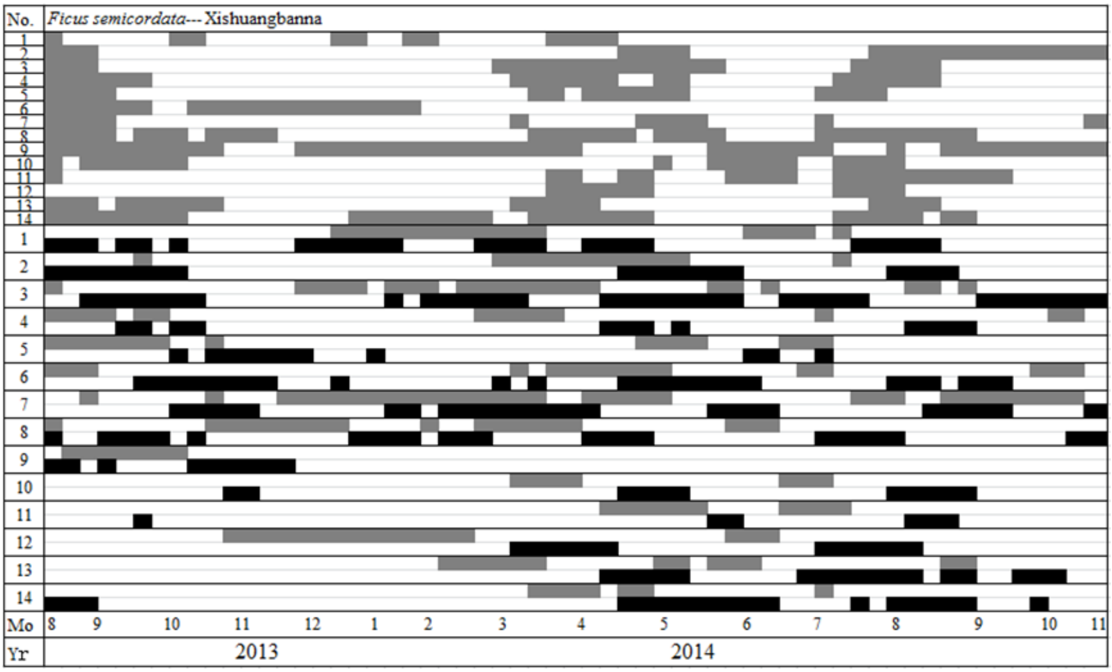

(a)

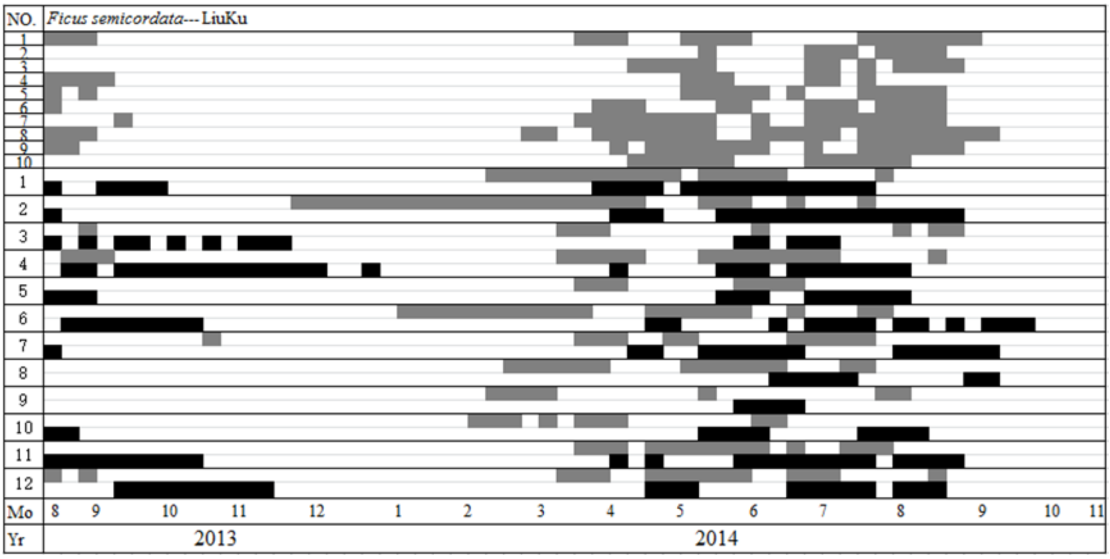

(b)

Supplementary Figure S6. Production of B phase (grey) and D phase (black-female plants only) syconia in *F. semicordata* in (a) Xishuangbanna and (b) in Liuku.

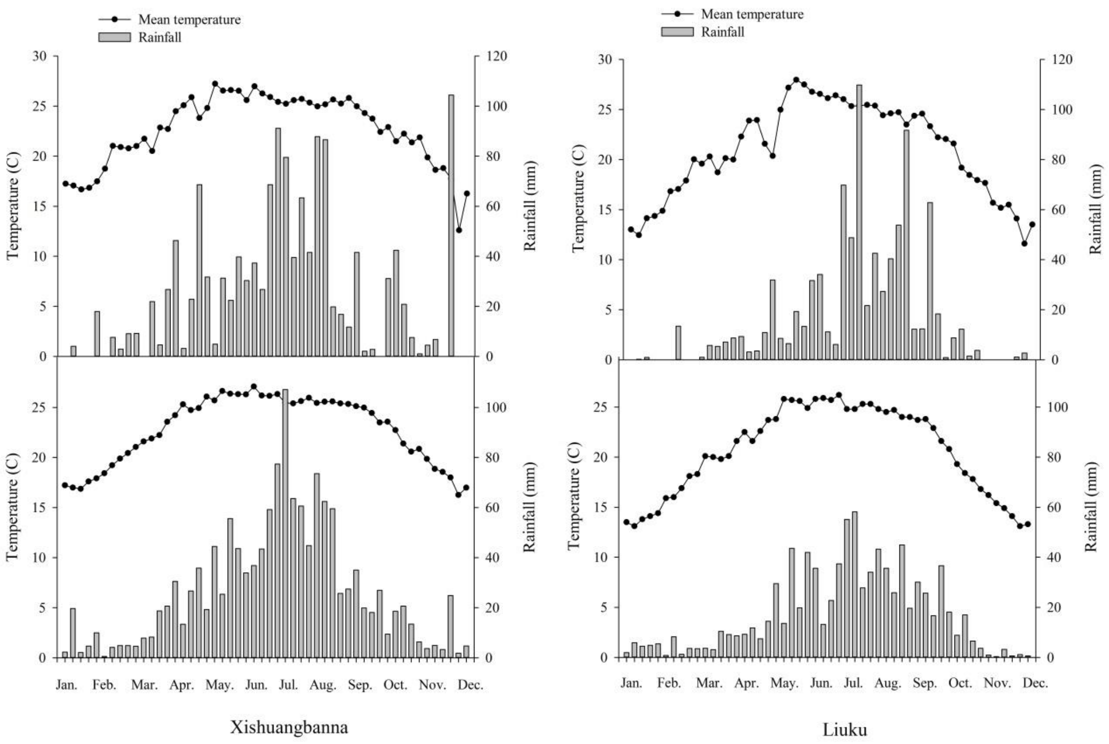

Supplementary Figure S7. Mean weekly temperature and rainfall at the nearest weather station during the study period (top row) and during the last 10 years (bottom row), in Xishuangbanna (left) and Liuku (right).

Supplementary Table S1. **Contents of the mature syconia of *Ficus altissima*, *Ficus racemosa* and *Ficus semicordata* (mean ± SD) and mean proportion of wasps (%).** For the dioecious *F. semicordata*: M male syconia, F female syconia.

Species & Sites	N of syconia	Season	Female flowers	Seeds	Female pollinators (%)	Cheaters (%)	Other NPFWs (%)
F. altissima<br>Xishuangbanna	183	Summer<br>Winter	534.5 ±12.3<br>569.2 ±17.6	178.0 ±12.0<br>136.9 ±12.5	128.7 ±9.4 (75.1%)<br>105.2 ±8.8 (63.8%)	20.9 ±7.1 (8.2%)<br>25.4 ±5.0 (16.3%)	18.1 ±3.3 (16.7%)<br>23.2 ±3.5 (19.9%)
F. altissima<br>Liuku	160	Summer<br>Winter	603.3 ±9.2<br>631.0 ±7.3	129.4 ±14.0<br>103.7 ±13.9	27.3 ±6.7 (17.7%)<br>67.1 ±9.0 (39.8%)	170.8 ±12.3<br>(79.2%)<br>90.7 ±8.8 (56.5%)	3.1 ±1.0 (3.0%)<br>6.2 ±1.9 (3.7%)
F. racemosa<br>Xishuangbanna	174	Summer<br>Winter	5283.0 ±164.9<br>5834.8 ±151.5	2079.3 ±175.9<br>1353.7 ±168.1	1012.9 ±83.2 (73.10%)<br>485.1 ±80.0 (45.65%)	0<br>0	165.6 ±11.4 (26.90%)<br>408.0 ±38.1 (54.35%)
F. racemosa<br>Liuku	165	Summer<br>Winter	3532.9 ±87.6<br>4090.9 ±52.7	589.8 ±78.0<br>451.3 ±68.6	67.5 ±11.8 (12.32%)<br>81.7 ±17.7 (13.84%)	0<br>0	326.4 ±16.7 (87.68%)<br>300.1 ±19.6(86.16%)
F. semicordata<br>Xishuangbanna	136 (M) + 98(F)	Summer<br>Winter	1932.7 ±31.5 (M)<br>2114.6 ±58.3 (M)	1504.6 ±72.2 (F)<br>1299.8 ±99.4 (F)	460.6 ±25.9 (62.85%)(M)<br>403.2 ±48.5 (84.01%)(M)	0<br>0	97.4 ±16.0 (37.15%)(M)<br>118.5 ±18.9 (15.99%)(M)
F. semicordata<br>Liuku	104 (M) + 106 (F)	Summer<br>Winter	2167.2 ±49.6 (M)<br>1637.5 ±44.6 (M)	1109.6 ±53.6 (F)<br>1333.9 ±64.3 (F)	145.9 ±30.2 (59.02%)(M)<br>26.1 ±4.2 (42.45%)(M)	0<br>0	70.7 ±9.0 (40.98)(M)<br>44.9 ±8.0 (57.55%)(M)

Supplementary Table S2. Non-pollinating fig-wasps in summer and winter crops of *Ficus altissima* (mean ± SD) and mean proportion of wasps (%).

Species	Season	Xishuangbanna (183 syconia)	Liuku (160 syconia)
Micranisa ralianga	summer	0.75 ± 0.26 (0.48%)	0.52 ± 0.19 (0.25%)
	winter	7.41 ± 1.21 (5.90%)	1.96 ± 0.69 (1.07%)
Micranisa sp.	summer	0.16 ± 0.09 (0.12%)	0.44 ± 0.26 (0.19%)
	winter	1.17 ± 0.28 (1.63%)	0.08 ± 0.06 (0.05%)
Walkerella sp.	summer	0.19 ± 0.08 (0.19%)	0.02 ± 0.02 (0.01%)
	winter	0.16 ± 0.08 (0.07%)	0
Sycoscapter sp.1	summer	0 (0%)	0
	winter	0.22 ± 0.11（0.15%）	0
Sycoscapter sp.2	summer	0.12 ± 0.07 (0.06%)	0
	winter	0.55 ± 0.20 (0.38%)	0
Sycoscapter sp.3	summer	0.65 ± 0.21 (0.35%)	0.85 ± 0.49 (0.32%)
	winter	4.11 ± 1.20 (2.78%)	2.68 ± 1.01 (1.47%)
Sycoscapter sp.4	summer	0.04 ± 0.03 (0.02%)	0.03 ± 0.03 (0.01%)
	winter	0.19 ± 0.09 (0.14%)	0.01 ± 0.01 (0.01%)
Philotrypesis sp.1	summer	1.95 ± 0.45 (1.59%)	0.05 ± 0.04 (0.02%)
	winter	2.03 ± 0.41 (1.30%)	0 (0%)
Sycobia sp.	summer	0 (0%)	0 (0%)
	winter	0.31 ± 0.13 (0.34%)	0 (0%)
Sycophilomorpha sp.	summer	10.46 ± 2.65 (10.45%)	0 (0%)
	winter	3.81 ± 1.79 (4.70%)	1.43 ± 1.28 (1.11%)
Acophila sp.	summer	0.34 ± 0.13 (0.20%)	0.11 ± 0.09 (0.07%)
	winter	0.48 ± 0.16 (0.57%)	0 (0%)
Genus indet	summer	0 (0%)	1.03 ± 0.76 (2.17%)
	winter	2.03 ± 2.03 (0.96%)	0 (0%)
Sycophila decatomoides	summer	2.77 ± 1.13 (2.78%)	0 (0%)
	winter	0.07 ± 0.05 (0.07%)	0 (0%)
Sycophila sp.1	summer	0.11 ± 0.08 (0.20%)	0 (0%)
	winter	0 (0%)	0 (0%)
Sycophila sp.2	summer	0.20 ± 0.15 (0.13%)	0 (0%)
	winter	0.02 ± 0.01(0.02%)	0 (0%)
Sycophila sp.3	summer	0 (0%)	0 (0%)
	winter	0.01 ± 0.01 (0.02%)	0 (0%)
Ormyrus sp.1	summer	0.04 ± 0.02 (0.05%)	0 (0%)
	winter	0.29 ± 0.11 (0.43%)	0 (0%)
Ormyrus sp.2	summer	0 (0%)	0 (0%)
	winter	0.19 ± 0.09 (0.25%)	0 (0%)
Ormyrus sp.3	summer	0.09 ± 0.07 (0.06%)	0 (0%)
	winter	0.13 ± 0.07 (0.18%）	0 (0%)

Supplementary Table S3. Non-pollinating fig-wasps in summer and winter crops of *Ficus racemosa* (mean ± SD).

Species	Season	Xishuangbanna (174)	Liuku (165)
Sycophaga testacea	summer	26.4 ± 3.2	67.7 ± 5.0
	winter	104.9 ± 15.5	99.1 ± 10.7
Sycophaga mayri	summer	81.4 ± 9.5	201.5 ± 13.5
	winter	229.5 ± 27.6	178.7 ± 16.4
Sycophaga agraensis	summer	23.5 ± 2.8	8.2 ± 2.1
	winter	14.1 ± 3.2	4.3 ± 1.8
Apocrypta westwoodi	summer	31.0 ± 4.8	45.0 ± 3.9
	winter	38.1 ± 4.9	10.8 ± 1.7
Apocrypta sp.	summer	3.4 ± 0.7	8.9 ± 1.3
	winter	21.4 ± 4.4	7.1 ± 0.9

Supplementary Table S4. Non-pollinating fig-wasps in summer and winter crops of *Ficus semicordata* (mean ± SD). All wasps were from syconia from female plants.

Species	Season	Xishuangbanna (136)	Liuku (104)
Sycophaga cunia	summer	18.0 ± 2.7	23.4 ± 5.5
	winter	16.7 ± 3.4	12.6 ± 2.6
Sycoscapter trifemmensis	summer	141.3 ± 12.9	37.7 ± 6.8
	winter	54.8 ± 13.4	26.7 ± 5.9
Philotrypesis dunia	summer	7.9 ± 1.6	9.5 ± 1.8
	winter	29.9 ± 13.1	5.6 ± 1.1
Apocrypta sp.	summer	21.3 ± 3.5	0.1 ± 0.1
	winter	14.2 ± 4.8	0
